# A hemodynamic model to guide blood pressure control during deliberate hypotension with sodium nitroprusside in children

**DOI:** 10.3389/fphar.2015.00151

**Published:** 2015-07-28

**Authors:** Jeffrey S. Barrett, Sarapee Hirankarn, Nick Holford, Gregory B. Hammer, David R. Drover, Carol A. Cohane, Brian Anderson, Erin Dombrowski, Tammy Reece, Anne Zajicek, Scott R. Schulman

**Affiliations:** ^1^Clinical Pharmacology and Therapeutics Division, The Children's Hospital of Philadelphia and Department of Pediatrics, University of Pennsylvania Medical SchoolPhiladelphia, PA, USA; ^2^Department of Pharmacology and Clinical Pharmacology and Anesthesia, University of AucklandAuckland, New Zealand; ^3^Department of Anesthesia, University of AucklandAuckland, New Zealand; ^4^Department of Anesthesiology, Perioperative and Pain Medicine, Stanford University School of MedicineStanford, CA, USA; ^5^Duke Clinical Research Institute, Duke University Medical CenterDurham, NC, USA; ^6^The Eunice Kennedy Shriver National Institute of Child Health and Human Development, National Institutes of HealthBethesda, MD, USA

**Keywords:** sodium nitroprusside, hemodynamics, models, biological, pediatrics, hypotension, controlled

## Abstract

Sodium nitroprusside (SNP) has been widely used to control blood pressure in infants and children. The goals of this analysis were to develop models that describe the hemodynamic response to SNP dosing in pediatric patients; examine sources of variation in dose-response, defining age, and size dependencies; and determine vulnerable populations or patient subtypes that may elicit dosing modifications. A multi-center, randomized, double-blinded, parallel-group, dose-ranging, effect-controlled study, followed by an open-label dose titration of an intravenous infusion of SNP was undertaken in 203 pediatric subjects, who required deliberate hypotension or controlled normotension during anesthesia. A total of 3464 MAP measurements collected from 202 patients during the study's blinded phase, including baseline measurements up to 6 min prior to the blinded were available for analysis. A population K-PD model was developed with a one-compartment model assumed for SNP. Size differences in CL and V of the effect compartment were described using theory-based allometry. An inhibitory sigmoidal E_max_ model was used to describe the effect of SNP. A power function of age was used to describe age-related differences in baseline MAP. A mixture model of two groups with low and high EC50 was used to explain variability in MAP response. Change in MAP was characterized by a linear disease progression slope during the blinded phase. In the final population model, CL and V increased with weight, and baseline MAP increased with age. The effect compartment half-life of SNP was 13.4 min. The infusion rate producing 50% of E_max_ (ER50) at steady state for high EC50, was 0.34 μg/kg/min and for low EC50 0.103 μg/kg/min. The K-PD model well-describes initial dosing of SNP under controlled circumstances; model-based dosing guidance agrees with current practice. An initial titration strategy supported via algorithm-based feedback should improve maintenance of target MAP.

## Introduction

Controlled hypotension, also referred to as deliberate hypotension, is a technique in general anesthesia in which a short-acting hypotensive agent is administered to reduce blood pressure and thus bleeding during surgery. The procedure facilitates surgery by making vessels and tissues more visible through reduced blood loss. While the use of general anesthetic agents or regional anesthesia is an alternative, management of controlled hypotension with sodium nitroprusside (SNP) (Degoute, 2007) has been common strategy in pediatrics for many years. Despite the usage, dosing guidance has been empirically-based and varies across institutions and practices. SNP is an effective hypotensive agent used in a variety of indications including controlled hypotension in children. Despite its widespread use, there is a paucity of information on the dose–response relationship. Due to rapid metabolism and chemical instability and thus a short half-life, the measurement of SNP plasma concentration for pharmacokinetic (PK) analysis has not been possible, despite numerous attempts to quantify the parent compound and metabolites (Pérez-Ruiz et al., 1991; Vesey et al., 1999). Initial dosing is based on patient weight with subsequent empirical adjustment of dose (infusion rate and duration) largely guided by the individual mean arterial pressure (MAP) response over time.

In support of the Best Pharmaceuticals for Children Act (BPCA), a multi-center, randomized, double-blinded, parallel-group, dose-ranging, effect-controlled study, followed by an open-label dose-titration of an intravenous infusion of SNP (Drover et al., 2015) has recently been completed. The study was in response to the written request authored by the Food and Drug Administration (FDA), pursuant to Section 505A of the Federal Food, Drug and Cosmetic Act (http://bpca.nichd.nih.gov/clinical/requests/Documents/sodium_nitroprusside.pdf). In addition to providing meaningful labeling for pediatric surgical patients receiving SNP, a model-based approach to characterize the relevant pharmacokinetic and pharmacodynamic (PKPD) response to SNP was advocated in the written request, particularly with the emphasis to provide dosing guidance. In the absence of an exposure biomarker (drug and/or metabolite concentration), the primary analysis was driven by dose–response characterization, where patient characteristics including indices of size and maturation, demographics, clinical conduct, and patient status were used as covariates to explore time-dependent changes in MAP as a function of the time course of dosing. Hemodynamic variables collected during this investigation included MAP, systolic, and diastolic blood pressure, and heart rate.

The objectives of the population-based PKPD analysis were to: develop model(s) that describe the hemodynamic response to SNP dosing in pediatric patients; examine sources of variation in the dose–response relationship, particularly defining age and size dependencies; determine vulnerable populations or patient subtypes that may elicit dosing modifications different from those for the mainstream population; and provide dosing guidance suitable for labeling, supported by simulations of the likely variation in MAP response. We present the details of the hemodynamic K-PD analysis herein, along with simulations based on the model that assess dosing considerations in children.

## Materials and methods

### Study design

The study (NICHD-2003-09-DR) was a multi-center, randomized, double-blinded, parallel-group, dose-ranging, effect-controlled study, followed by an open-label, dose-titration of an intravenous infusion of SNP in 203 pediatric subjects (birth to 17 years of age). These subjects required deliberate hypotension or controlled normotension for an anticipated duration of at least 2 h^1^. The design and support for the study resulted from the BPCA. The details of the study design and initial dosing and subsequent dosing titration are presented in a companion manuscript (Drover et al., 2015).

Any patient who had the first blood sample for PK analysis collected during the blinded study drug administration period or who discontinued blinded study drug was considered complete for analysis because:

- MAP fell below 50 mm Hg (40 mm Hg for neonates), or
- Heart rate exceeded the age-adjusted maximum (Drover et al., 2015), or
- An adverse event occurred that was judged possibly or probably related to the study drug.

At least one hundred (≥50%) of these patients were targeted to be pre-pubertal (< Tanner stage 3), with at least 50% of them being neonates (post-natal age less than 1 month or infants (from 1 month to < 2 years of age). In order to qualify for inclusion, neonates were required to be full-term gestation and have a weight of ≥2.5 kg.

Briefly, after their anesthesia and measurement of vital signs were stabilized, subjects began the blinded study drug administration period (dose-ranging). During this phase, subjects were randomized in equal proportions to receive SNP at one of four doses: 0.3, 1.0, 2.0, or 3.0 μg/kg/min, administered as a continuous intravenous infusion for up to 30 min. Dose titrations were made during the double-blind study drug administration period to minimize the potential risk of excessive drop in blood pressure to study participants. During titration to the targeted rate, the investigator was requested to keep the previously titrated rate constant for the remainder of the 30 min, if clinically acceptable. Following 30 min of blinded study drug administration, an open-label titration of the study drug was initiated until blood pressure control was achieved (Drover et al., 2015). Dose increments of 0.1–1.0 μg/kg/min were utilized with the desire to control target MAP (effect-control dosing). The total duration of observation was up to 180 min during this phase, and rescue medication was allowed. The follow-up period was to end 24 h after completion of the study drug administration phase or hospital discharge, whichever occurred first.

### Drug administration

Infusion pumps capable of reliable delivery at low infusion rates (to 0.1 mL/h) were used to administer SNP. All syringe pumps had free flow protection. Captured syringe type infusion pumps had been internally calibrated for accuracy by the manufacturer and verified by each site as part of an equipment management program. Quality-assurance checks were performed periodically according to manufacturer specifications. Microbore low-compliance tubing, with volumes of approximately 1 mL or less was used. The infusate syringe was covered with an amber or opaque plastic wrap to protect the drug from light.

The study drug was infused via either a dedicated peripheral intravenous catheter or a dedicated lumen of a multi-orifice, central-venous catheter. Catheters were chosen to minimize dead space in order to ensure rapid, accurate delivery of the drug to the subject. The study drug was administered along with a carrier fluid such that the carrier fluid was infusing at a minimum of 10 mL/h, further eliminating the dead space issue. Carrier, blinded drug, and open-label drugs were attached and flushed through a 2-tailed T-piece connector such that there was no time delay in switching from blinded to open-label infusion, as well as to add no further dead space to the tubing before the invasive catheter.

### PKPD data

Due to low concentrations resultant from a short half-life, rapid metabolism, and chemical instability, the measurement of SNP concentration in plasma was not possible (Kazim et al., 1996). Analyses of cyanide, thiocyanate, methemoglobin, lactic acid, and arterial blood-gas analysis with co-oximetric analysis were performed throughout the trial to indirectly monitor sodium nitroprusside exposure. The measurement of cyanide in plasma employing a classical UV bioanalytical method was provided by National Medical Services, Inc. (NMS Labs). All of the cyanide specimens collected had measured values that were below the assay limit of detection (0.10 μg/mL).

The mean arterial pressure (MAP) was the primary response and declared efficacy endpoint investigated in this trial. Other hemodynamic response variables (systolic and diastolic blood pressure and heart rate) were also measured. This analysis is focused on MAP response.

The MAP was recorded:

- Immediately prior to and every 15 min after the initiation of anesthesia until the blinded study drug was initiated.
- Immediately prior to and every 2 min for 30 min after the initiation of the blinded study drug.
- If the open-label study drug did not start immediately after the end of the blinded study drug infusion, every 2 min for 10 min, then every 5 min for 20 min, and then every 15 min until open-label infusion started.
- Immediately prior to and every 2 min for 10 min after the initiation of the open-label study drug and any rate change of open-label and then every 5 min throughout the duration of the study drug administration.
- Immediately before, and every 2 min for the first 10 min after the study drug was discontinued and then every 5 min for the next 20 min, for a total of 30 min after discontinuation.
- Every 2 min for 10 min following the start of an adverse event that was possibly or probably related to the study drug, followed by every 5 min for the duration of the adverse events (AE), then every 5 min for 30 min upon resolution of the adverse event.

### Analysis datasets

The dataset from the blinded phase was used for model development, and the dataset from the open-label phase was used for model evaluation (Mentre and Escolano, 2006; Brendel et al., 2007). Differences between the two phases with regards to the analysis datasets are aligned with the objectives of each study phase.

Only the MAP baseline values measured within 6 min prior to the initiation of the blinded phase were included in model construction. While creating analysis datasets, certain subject and/or MAP measurement records were excluded for one or more of the following reasons: (1) there was no dose information, (2) the time was not recorded, (3) data were deemed inaccurate by the investigator.

All data preparation and presentation were performed with either the SAS software, version 9.2, or R software, version 2.8.1. The analysis was completed using NONMEM software, version 6.2 (ICON Development Solutions).

### Model assessment and workflow

The minimum value of the objective function (OFV), computed in NONMEM for each analysis, was used to evaluate and compare models in a hierarchical manner. The difference in OFV was considered to be chi-square distributed for the purpose of hypothesis-testing. The goodness-of-fit of each NONMEM analysis was assessed by the examination of the following criteria: (a) agreement in scatterplots of predicted and individual predicted values vs. measured values; (b) lack of trend or pattern in scatterplots of residuals and weighted residuals vs. predicted values; and (c) lack of trend or pattern in scatterplots of residuals and weighted residuals vs. predicted values.

The population analysis for SNP performed on the blinded phase dataset included all baseline values in the following sequence of steps: MAP baseline value assessment, structural K-PD model development, random-effect model development, final parameter-estimation, and model evaluation. The estimation methods, including the first-order conditional estimation method (FOCE) and the FOCE with interaction method (FOCE-I), were evaluated at various stages of the development process. NONMEM analyses were performed on a Microsoft Windows (XP) operating system. The g77 (version 0.5.25) FORTRAN compiler was used to create NONMEM executable files. Wings for NONMEM (Holford, 1996) was used to produce bootstrap results.

### Structural K-PD model development

A K-PD model approach (Jacqmin et al., 2007; Holford, 2010) was utilized for this analysis, given the lack of plasma concentration data. With this approach, the MAP response as a function of biophase exposure is defined by the time course of drug input (dose). SNP concentrations are predicted on the basis of a one-compartment model representing the effect compartment. The effect compartment was assumed to have a nominal volume of distribution (V) of 1 L/70 kg. The clearance of the effect compartment (CL) was derived based on this assumed volume. The drug concentration in the effect compartment (Ce) is estimated based on the assumption of an instantaneous relationship between Ce and the predicted response (MAP in this case). Allometric scaling on effect-compartment parameters, the clearance (CL) and the volume of distribution (V), were employed to account in a biologically plausible fashion for differences in the time course of Ce associated with weight (Anderson and Holford, 2008; Holford, 2010) based on allometry theory.

### PD model

An inhibitory sigmoidal E_max_ model was used initially. Logistic transformation was employed to constrain the fractional change in E_max_, relative to E_0_, to be from 0 to 1. The examination of the parameter distributions of the starting model revealed that there were two subpopulations of EC_50_ and E_max_. A mixture model on EC_50_ fitted the data better than the mixture model on E_max_. Therefore, the mixture model on EC_50_, low EC_50_, and high EC_50_ was selected for further model development. Regarding the natural disease-progression model, the use of a linear model improved the model fit. Age was associated with higher baseline MAP. PD equations of the model are provided below.

(1) $$\begin{array}{l} {\text{MAP}}_{\text{ij}}=\{\begin{array}{l} \;{\text{S}}_{\text{0},\text{ i}}{-\text{E}}_{\text{max},\text{i}}•{\text{C}}_{\text{e},\text{ ij}}^{\gamma }\text{/}(\text{High}\_{\text{EC}}_{\text{50},\text{i}}^{\gamma }+{\text{C}}_{\text{e},\text{ ij}}^{\gamma })\\ \;{\text{S}}_{\text{0},\text{ i}}{-\text{E}}_{\text{max},\text{i}}•{\text{C}}_{\text{e},\text{ ij}}^{\gamma }\text{/}(\text{Low}\_{\text{EC}}_{\text{50},\text{i}}^{\gamma }+{\text{C}}_{\text{e},\text{ ij}}^{\gamma }) \end{array}\\ \;+\;\alpha •\text{Time} \end{array}$$

(2) $$\begin{array}{rcl} {\text{S}}_{0,\;\text{i}} & = & {\text{S}}_{0,\;\text{i}}{\left(\frac{{\text{Age}}_{\text{i}}}{\text{Median}\;\text{Age}}\right)}^{\text{PwrAge}} \end{array}$$

where MAP_ij_, the MAP at *j*th time in the *i*th individual; S_0, i_, the baseline MAP in the *i*th individual; E_max,i_, maximal effector maximal decrease of MAP in the *i*th subject; C_e,ij_, SNP effect compartment concentration at *j*th time in the *i*th subject; EC_50,i_, nominal SNP concentration or SNP concentration in effect compartment in the *i*th subject that leads to 50% of the effect; γ, Hill coefficient for the sigmoidal E_max_ function; Age_i_, the post-natal age for the *i*th subject; PwrAge, the power function of age.

Between-subject variability (BSV) of V, CL, S_0_, Low EC_50_, High EC_50_, and E_max_ was modeled using an exponential random-effect model. The linear disease-progression slope was based on a proportional random-effects model permitting positive or negative individual slope values (Chan and Holford, 2001). A non-parametric bootstrap involving 1000 replicates was used to create the bootstrap distribution of the parameters. The bootstrap average and 95% confidence interval for each parameter was estimated from the bootstrap distribution.

### Model evaluation

A visual predictive check (VPC), based on the premise that a model and parameters derived from the observed data set should produce simulated data similar to the original observed data, was utilized to evaluate the adequacy of the final model. One hundred replicates of the original dataset were simulated, based on the final model, and a 95% confidence interval, based on simulated datasets, was computed for the 5th, 50th, and 95th percentiles. Percentiles of observed and predicted values were constructed in order to compare the ability of the model to predict the median and 90% interval.

### Simulations

Simulations were performed to examine dosage regimens of SNP that mimic typical patient settings and clinical objectives. Two clinical scenarios were considered:

#### Scenario 1

The objective of this simulation was to find doses to achieve the MAP reduction of a certain value (10 and 15 mm Hg) within a clinically-relevant time frame (assumed 5 min). Hypothetical patients with different age/weight designation were included. Tanner age strata were considered, and a median age/weight for each stratum was used when considering hypothetical patients. The median age/weight for each stratum was calculated from the data used for the model development. Since there are two subpopulations identified in the model, more sensitive to SNP (low EC_50_) and less sensitive (high EC_50_) groups were examined as well.

#### Scenario 2

The objective of this simulation was to the find an initial dose to reduce MAP to the certain target value (assumed 60 mm Hg) within 3 min, and then to identify a second dose to maintain MAP at that value for 30 min.

Simulations ignoring variability (deterministic) and incorporating variability (stochastic) were performed. Deterministic simulations were based on the population predicted values from the final model, whereas stochastic simulations (not shown), based on 100 replicates per scenario, used the full variance-covariance matrix.

## Results

### Analysis dataset

A total of 203 patients enrolled in the study and total of 3038 MAP measurements obtained from 202 patients were available for the model-building analysis. For the open-label portion of the study, a total of 10,671 MAP measurements were available from 203 subjects. By applying the same criteria (as for the blinded study data), a total of 10,303 MAP measurements were available from 197 patients for the qualification/validation analysis. Summary statistics of subject demographic characteristics, dosing data, and MAP measurements are summarized in Table 1.

**Table 1:** **Summary statistics of variables included in model development dataset**.

**Variable**	**Continuous variables**			
	**Mean**	**Median**	**Min**	**Max**
**DOSE VARIABLES**				
Dose rate (μg/kg/min)	0.87	0.66	0.09	3.04
Infusion duration (min)	8.61	5	1	30
**PD VARIABLE**				
MAP (mm Hg)	69.4	68	29	143
**DEMOGRAPHIC VARIABLES**^a^				
Age (y)	8.83	11.3	0.01	17.01
Weight (kg)	33.8	31.6	2.8	112.2
	**Categorical variables N (%)**			
Gender				
Male	73 (36)			
Female	129 (64)			
Race				
American Indian	13 (6.4)			
Asian	7 (3.5)			
Black	16 (7.92)			
Pacific Islander	1 (0.5)			
White	164 (81.2)			
Other	1 (0.5)			
Ethnicity				
Hispanic	47 (23.3)			
Not Hispanic	155 (76.7)			
FDA Age Category^b^				
Neonates	4 (2)			
Infants	51 (25.2)			
Children	47 (23.3)			
Adolescents	100 (49.5)			
Development strata for enrollment				
Birth to < 30 days	4 (2)			
30 days to < 2 years	51 (25)			
2 to < 6 years	12 (6)			
6 yrs to Tanner Stage III	43 (21)			
Tanner Stage III to < 17 years	92 (46)			

a *No of Subjects = 202*.

b *Per FDA Guidance (General Clinical Pharmacology Considerations for Pediatric Studies for Drugs and Biological Products: Guidance for Industry, www.fda.gov/downloads/drugs/guidancecomplianceregulatoryinformation/guidances/ucm425885.pdf)*.

### Population K-PD model

**Figure 1:**
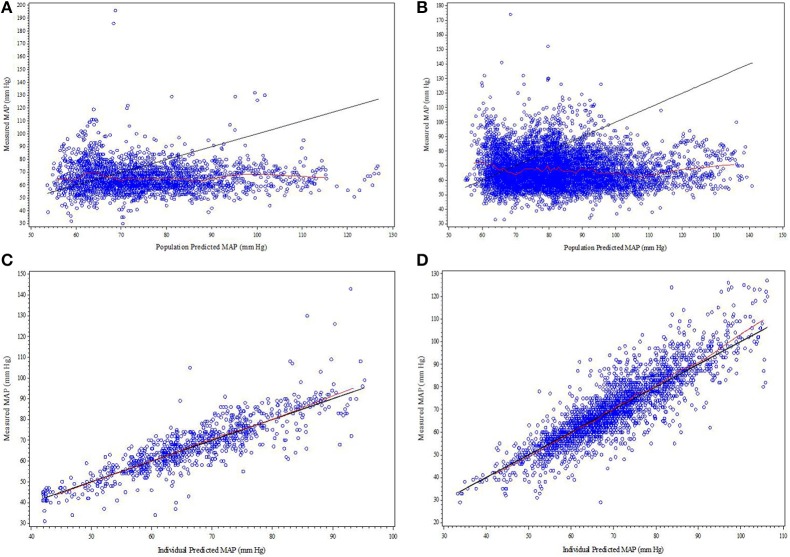
**(A–D)** Diagnostic plots from final SNP K-PD hemodynamic model constructed from blinded phase data for low and high MAP response groups: **(A)** Low EC_50_ population-predicted vs. observed, **(B)** High EC_50_ population-predicted vs. observed, **(C)** Low EC_50_ individual-predicted vs. observed, and **(D)** High EC_50_ individual-predicted vs. observed.

**Figure 2:**
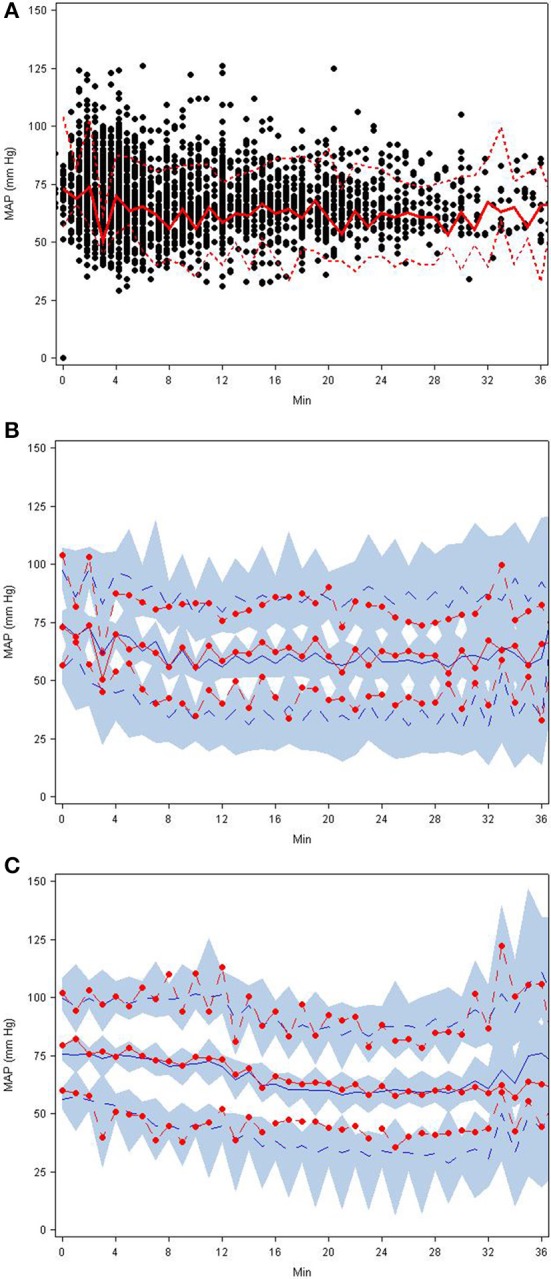
**(A–C)** Visual predictive check plots based on the final SNP K-PD hemodynamic model: **(A)** observed MAP response with 5th, 50th, and 95th percentiles, **(B)** predictions and prediction intervals for low EC_50_ group, and **(C)** predictions and prediction intervals for high EC_50_ group.

Parameter estimates and standard errors for the final population K-PD model are provided in Table 2. The infusion rate producing 50% of E_max_ (ER_50_) at steady state was calculated from the product of the population standard values of EC_50_ and CL. All parameters were generally well-estimated with reasonable precision (RSE < 25%), except for the between subject variability of high EC_50_ (RSE = 74.3%), and low EC_50_ (RSE = 72.0%). The goodness-of-fit plots for final model (Figures 1, 2) overall indicated that there were no strong biases in the fit of this model to the data.

**Table 2:** **Parameter estimates and standard errors for the final K-PD model**.

**Parameter**	**Population parameters**			**Magnitude of BSV**	
	**Estimate**		**Bootstrap %RSE**	**Bootstrap average (%CV)^d^**	**Bootstrap %RSE**
	**Model^a^**	**Bootstrap average^b^**			
V (L/70 kg)	1 (FIXED)	1 (FIXED)	NA	82.0	7.6
CL (L/h/70 kg)	3.12	3.13	9.6	62.7	16.1
S_0_(mm Hg)	76	76	1.0	13.2	5.4
Power of age on S_0_	0.0333	0.032	19.2		
E_max_ (mm Hg)^e^	22.65	21.9	7.6	76.5	13.7
Probability of high EC_50_ subpopulation	0.699	0.701	8.8		
EC_50_ (μg/L)—High	460	458	10.3	10.8	74.3
EC_50_ (μg/L) —Low	138	104.3	21.6	22.1	72
Hill coefficient	7.23	7.27	11.2		
Disease-progression slope (mm Hg)	12.3	11.0	19	211	19.3
ER_50_ (mg/h/70 kg)—High^c^	1.435	0.33	24.1		
ER_50_ (mg/h/70 kg)—Low^c^	0.431	1.42	14.2		
Residual variability (%CV)	32.9	33.2	3.7		

a *The parameter estimates were obtained from final model using NONMEM*.

b *The estimates were obtained from the bootstrap distribution*.

c *ER_50_ at steady state was calculated from the product of nominal EC_50_ and CL*.

d *CV% is the apparent coefficient of variation obtained from the square root of the estimate of the variance of between subject variability*.

e *BSV of E_max_ used a logistic transformation of the random effect to constrain the value between 0 and E_0_*.

The final covariate models for computing CL, V, and S_0_ of the final population model are provided in Equations (3–5) below.

(3) $$\begin{array}{rcl} {\text{CL}}_{\text{i}}\;\left(\text{L}∕\text{h}∕70\text{kg}\right) & = & 3.13∙{\left(\frac{{\text{WT}}_{\text{i}}}{70}\right)}^{0.75} \end{array}$$

(4) $$\begin{array}{rcl} {\text{V}}_{\text{i}}\left(\text{L}∕70\text{kg}\right) & = & 1∙\left(\frac{{\text{WT}}_{\text{i}}}{70}\right) \end{array}$$

(5) $$\begin{array}{rcl} {\text{S}}_{0,\;\text{i}}\left(\text{mmHg}\right) & = & 76∙{\left(\frac{{\text{Age}}_{\text{i}}}{11.33}\right)}^{0.0338} \end{array}$$

The calculated effect compartment half-life of SNP was 13.4 min. The ER_50_ for groups with low and high EC_50_ was 0.103 and 0.34 μg/kg/min. ER_50_ is an infusion rate producing 50% of E_max_ at steady state. It was calculated from the product of nominal EC_50_ and CL. MAP increased over time, independent of SNP exposure. Baseline MAP (S_0_) increased with post-natal age. Residual variability was estimated to have a co-efficient of variation of 33.2%.

### Model evaluation

**Figure 3:**
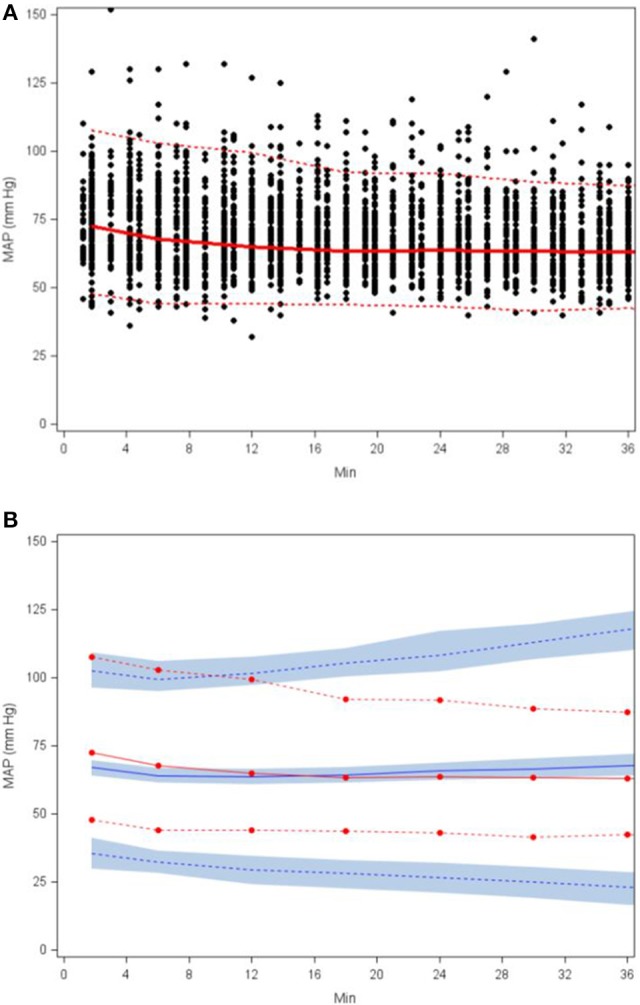
**Scatterplot of observed MAP with 5th, 50th, and 95th percentiles (A) and visual predictive check based on applying the final SNP K-PD hemodynamic model to the open (unblinded) phase MAP response (B)**.

The goodness-of-fit plots from the application of final model to the open-label phase data are presented in Figures 1A–D. The population predictions were high relative to the observed values. The visual predictive check shown in Figures 2A–C adequately describes the median and 95th percentile of observed MAP during the double-blind phase. The 5th percentile of predicted MAP was lower than observed at later times, likely due to homeostatic responses produced by low MAP, leading to tolerance. Figures 3A–B shows the application of the final model constructed from the blinded phase data to the open phase data (Figure 3A) and the visual predictive check applied to the open phase data (Figure 3B). Deviations from the extremes are most likely resultant from the unaccounted for (in the blinded phase model) procedures permitted during the open-label phase.

### Simulation scenarios

#### Simulation 1

**Figure 4:**
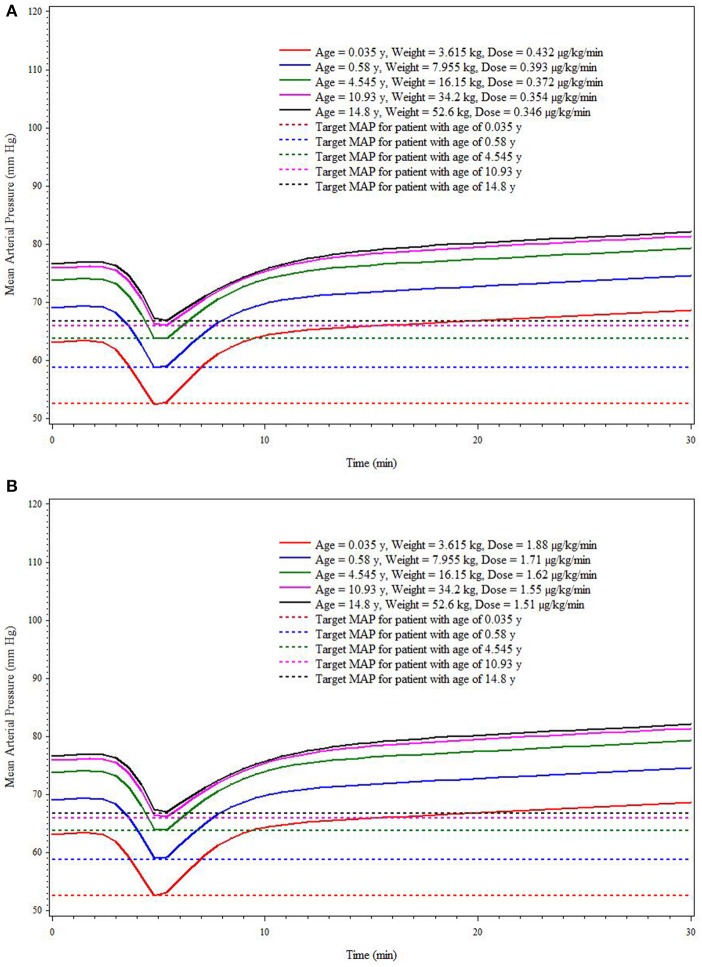
Simulation-based (deterministic) dose^*^ prediction for Low (A) and High (B) EC_50_ response groups to achieve target MAP across age strata (targeted reduction of 10 mm Hg within 5 min).

This simulation was based on five hypothetical patients (age/weight: 0.035 years/3.615 kg, 0.58 years/7.955 kg, 4.545 years/16.15 kg, 10.93 years/34.2 kg, and 14.8 years/52.6 kg) receiving a single administration of SNP. The simulation results reflect the median weight associated with each of the 5 age strata in the current trial. Due to the association of age with baseline MAP, the baseline MAP values are necessarily different for each hypothetical patient. The baseline MAP values for hypothetical patients of ages 0.035, 0.58, 4.545, 10.93, and 14.8 years are 63, 69, 74, 76, and 77 mm Hg, respectively. Figures 4A,B shows the MAP time course based on SNP dosing required to reduce MAP 10 mm Hg (Figure 4A) and 15 mm Hg (Figure 4B) for each hypothetical patient. For the low EC_50_ subpopulation, the doses needed for the 10 mm Hg reduction of MAP for hypothetical patients are 0.432, 0.393, 0.372, 0.354, and 0.346 μg/kg/min, respectively. For the high EC_50_ subpopulation, the doses needed for the 10 mm Hg reduction of MAP for hypothetical patients are 1.88, 1.71, 1.62, 1.55, and 1.51 μg/kg/min, respectively. For the low EC_50_ subpopulation, the doses needed for the 15 mm Hg reduction of MAP for hypothetical patients are 0.537, 0.465, 0.427, 0.415, and 0.407 μg/kg/min, respectively. For the high EC_50_ subpopulation, the doses needed for the 10 mm Hg reduction of MAP for hypothetical patients are 2.38, 2.01, 1.86, 1.77, and 1.73, respectively. These simulated dosing requirements are summarized in Table 3.

**Table 3:** **Dosing recommendation: single SNP infusion of 5 min**.

**Age (y)/Weight (kg)**	**Baseline MAP (mm Hg)**	**Target MAP reduction (mm Hg)**	**Low EC_50_ (μg/kg/min)**	**High EC_50_ (μg/kg/min)**
0.035/3.615	62.6	10	0.432	1.88
0.58/7.955	68.8	10	0.395	1.72
4.545/16.15	73.7	10	0.372	1.62
10.93/34.2	75.9	10	0.354	1.55
14.8/52.6	76.7	10	0.346	1.51
0.035/3.615	62.6	15	0.537	2.38
0.58/7.955	68.8	15	0.465	2.01
4.545/16.15	73.7	15	0.427	1.86
10.93/34.2	75.9	15	0.415	1.77
14.8/52.6	76.7	15	0.407	1.73

#### Simulation 2

**Figure 5:**
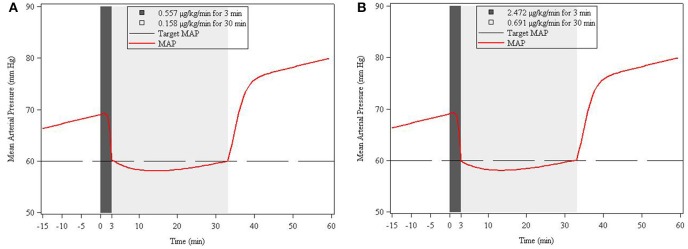
Simulation-based (deterministic) two-stage infusion dose^*^ prediction for Low (A) and High (B) EC_50_ response to achieve a target MAP of 60 mm Hg within 3 min and maintaining this level for 30 min. The simulation is based on the response in a representative (virtual) patient of 7 months weighing 8 kg. Shaded areas refer to infusion durations.

The results from the staggered dosing simulation were based on the same five hypothetical patient characteristics as in simulation 1. For convenience, in Figures 5A,B, we have shown only the MAP response in a representative patient of 7 months weighing 8 kg under both low and high EC_50_ conditions. Target MAP for all hypothetical patients was assumed to be the same (60 mm Hg). In all cases, the first dose was needed to get to the target MAP within 3 min, and the second dose was needed to maintain MAP at the target for 30 min. For the low EC_50_ subpopulation, the first dose for 7 month (0.58 years) patient was 0.566 μg/kg/min and the second dose was 0.158 μg/kg/min. For the high EC_50_ scenario, the first dose was 2.472 μg/kg/min, and the second dose was 0.691 μg/kg/min. Table 4 contains the summary of simulated dosing requirements for the staged infusion regimens for all 5 age strata for both low and high EC_50_ subpopulations.

**Table 4:** **Dosing recommendation: sequential SNP infusions of 5 and 30 min**.

**Age (y)/Weight (kg)**	**Baseline MAP (mm Hg)**	**Target MAP reduction (mm Hg)**	**Low EC50 First/Second infusion (μg/kg/min)**	**High EC50 First/Second infusion (μg/kg/min)**
0.035/3.615	62.6	2.6	0.479/0.164	2.118/0.718
0.58/7.955	68.8	8.9	0.577/0.158	2.472/0.691
4.545/16.15	73.7	13.7	0.617/0.163	2.683/0.722
10.93/34.2	75.9	15.9	0.635/0.195	2.778/0.828
14.8/52.6	76.7	16.7	0.640/0.206	2.744/0.951

## Discussion

A hemodynamic model for MAP response to intravenous infusion of SNP for the purpose of controlled intraoperative hypotension in children is presented. Given the absence of concentration data, a population kinetic-pharmacodynamic (K-PD) model was employed to investigate the relationship between SNP doses, time, and MAP response. The final model fitted the data obtained during the blinded phase following SNP administration including the MAP baseline measurements, reasonably well.

The over-prediction of MAP in the open blind phase reflects the use of procedures to change MAP not included in the model (based on the blinded phase data) used to make predictions. Several factors, such as pain (e.g., at the time of surgical incision of other manipulation), anesthesia, and excitement, may affect blood pressure. These factors were not recorded and therefore not accounted for in the model.

Most importantly, the parameter estimates seem to be plausible, given the assumed pharmacokinetic attributes of SNP. Specifically, the estimated effect compartment half-life is consistent with the extensive and rapid metabolism appreciated for SNP (Przybylo et al., 1995; Kazim et al., 1996). Likewise, the confirmation of baseline MAP increase with post-natal age is consistent with physiologic expectations. Baseline MAP values are most likely affected by the anesthesia at the beginning of the blinded phase.

In clinical practice, the SNP dose is initially based on patient weight; dose recommendations are based on weight because of the expected association of weight with SNP CL and V (Sinaiko, 1996; Tobias, 2002). It should be appreciated that the relationship between clearance and weight is not linear, however. As clearance is most important for maintaining concentrations, changes in weight with age necessitate that a fixed dose per kg cannot be used for all age groups. This is a fundamental property of theory-based allometry that was used in this analysis. The allometric scaling of effect-compartment clearance is expected to scale with an exponent of ¾ reflecting not only metabolism but also blood flow-dependent distribution to tissues.

Efforts were made to identify a maturation process associated with age that might be reflected in the apparent effect-compartment clearance. Despite the inclusion of infants and neonates in the patient population, there was no evidence for a change in clearance with age independently of size. We speculate this is because the chemical breakdown of SNP is not dependent on liver or kidney size, and thus there will be no maturation of metabolism associated with growth of these organs.

Although SNP is an old drug and its use is widespread, there is no accepted guidance for SNP dosing; labeling statements regarding dosing guidance for 5 age groups were derived from simulations using the final model. Nevertheless, as the model was developed in pediatric patients under anesthesia, parameter estimates (S_0_ and E_max_) in other settings may vary due to the influence of concomitant medications and procedures.

It is important to acknowledge that we have identified two groups of patients—those with a low C_50_ who need a low dose and those with a high C_50_ who need a higher dose. We were unable to distinguish any way to predict these two levels of sensitivity, on the basis of study data collected. A prudent approach is to assume all patients have low C_50_, then adjust dose higher if the response is not as expected.

Based on the simulation exercise, the reduction in MAP following SNP administration can be managed with a small number of dose adjustments. While hemodynamic improvement should be considered in conjunction with other meaningful clinical outcomes when assessing the overall efficacy of SNP, it is clear that a model-based approach may improve the tendency to “over-adjust” or too frequently titrate SNP in response to real time MAP (Spielberg et al., 2014). Parallel efforts to discriminate superior dosing practices in this investigation revealed much variability in dosing practice, with hemodynamic control well-outside of anticipated patient management expectations (Spielberg et al., 2014).

Indices of toxicity were not included in the analysis. Incidence of toxicity in this study was actually quite low (Drover et al., 2015). Similarly, as suggested doses from simulations are based on achievement of MAP targets, they may not reflect optimal doses, given the absence of toxicity information. Recently approved dosing guidance provided in the Federal Register states that “a starting dose of 0.3 μg/kg/min is reasonable,” based on the pooled dataset (HHS FDA, 2014). Indeed, based on current clinical practice, this is certainly a reasonable starting point and does bisect model-based recommendations. Hopefully, future evaluations will consider the clinical benefit of a more effectively managed patient (greater percentage of time within an acceptable target MAP range) as opposed to an empirical-based standard of care.

## Funding

NO1-HD-4-3385 (NICHD, Rockville, Maryland).

### Conflict of interest statement

The authors declare that the research was conducted in the absence of any commercial or financial relationships that could be construed as a potential conflict of interest.

## Footnotes

^1^Study Protocol Number: NICHD-2003-09-DR (NITPIC 1.0). Phase II, A Multicenter, Randomized, Double-blinded, Parallel Group, Dose-ranging, Effect-controlled Study to Determine the Pharmacokinetics and Pharmacodynamics of Sodium Nitroprusside in Pediatrics Subjects. 2004.
